# Investigating Expression Dynamics of miR-21 and miR-10b in Glioblastoma Cells In Vitro: Insights into Responses to Hypoxia and Secretion Mechanisms

**DOI:** 10.3390/ijms25147984

**Published:** 2024-07-22

**Authors:** Hanna Charbit, Iris Lavon

**Affiliations:** Leslie and Michael Gaffin Center for Neuro-Oncology, Agnes Ginges Center for Human Neurogenetics, Department of Neurology, Hadassah Medical Center, Faculty of Medicine, The Hebrew University of Jerusalem, Jerusalem 9112002, Israel

**Keywords:** glioblastoma, miR-21, miR-10b, hypoxia, VEGF alpha, bevacizumab

## Abstract

Glioblastoma poses significant challenges in oncology, with bevacizumab showing promise as an antiangiogenic treatment but with limited efficacy. microRNAs (miRNAs) 10b and 21 have emerged as potential biomarkers for bevacizumab response in glioblastoma patients. This study delves into the expression dynamics of miR-21 and miR-10b in response to hypoxia and explores their circulation mechanisms. In vitro experiments exposed glioma cells (A172, U87MG, U251) and human umbilical vein endothelial cells (HUVEC) to hypoxic conditions (1% oxygen) for 24 h, revealing heightened levels of miR-10b and miR-21 in glioblastoma cells. Manipulating miR-10b expression in U87MG, demonstrating a significant decrease in VEGF alpha (VEGFA) following miR-10b overexpression under hypoxic conditions. Size exclusion chromatography illustrated a notable shift towards miR-21 and miR-10b exosomal packaging during hypoxia. A proposed model suggests that effective bevacizumab treatment reduces VEGFA levels, heightening hypoxia and subsequently upregulating miR-21 and miR-10b expression. These miRNAs, released via exosomes, might impact various cellular processes, with miR-10b notably contributing to VEGFA level reduction. However, post-treatment increases in miR-10b and miR-21 could potentially restore cells to normoxic conditions through the downregulation of VEGF. This study highlights the intricate feedback loop involving miR-10b, miR-21, and VEGFA in glioblastoma treatment, underscoring the necessity for personalized therapeutic strategies. Further research should explore clinical implications for personalized glioma treatments.

## 1. Introduction

Glioblastomas, the most lethal form of brain tumors, present a significant challenge in oncology due to their aggressive nature and the lack of effective treatment options. The emergence of targeted therapies, such as bevacizumab, offers a novel intervention strategy. Bevacizumab, a monoclonal antibody targeting vascular endothelial growth factor (VEGF) alpha (VEGFA), disrupts tumor angiogenesis [1].

The inadequate brain vasculature in glioblastoma results in the formation of hypoxic gradients within the tumor, a common occurrence in the tumor microenvironment (TME) [2]. Hypoxia triggers significant physiological changes, impacting tumor behavior, including cellular metabolism and immune response alterations [3,4]. Angiogenesis, primarily mediated by the hypoxia-inducible factor (HIF)-1 pathway and VEGF expression, is a significant outcome of hypoxia in glioblastoma [5]. This upregulation of VEGFA leads to new blood vessel formation, which is crucial for tumor growth, and a higher microvessel density correlates with poorer patient survival [6,7,8]. Initially, bevacizumab showed promise in treating glioblastoma by disrupting this angiogenesis [1]. However, it faces challenges, as clinical trials often show initial improvements in progression-free survival followed by tumor recurrence and resistance, limiting overall survival benefits [9,10]. Hypoxia triggers adaptive mechanisms that may contribute to this resistance [11]. Cancer cells in hypoxic regions adopt a migratory and invasive phenotype, invading healthy tissue and “co-opting” nearby blood vessels to secure oxygen and nutrients, thereby fueling tumor progression [12,13].

MicroRNAs (miRNAs) play a crucial role in adapting to hypoxia by modulating gene expression and metabolic pathways [14]. Their rapid production, compact size, and reversible regulation make miRNAs efficient regulators in hypoxia-related processes [15].

Previous studies have identified several hypoxia-regulated miRNAs (HRMs) with altered expression in response to hypoxia. MiR-373, miR-372, miR-155, miR-210, and miR-10b show increased expression under hypoxia [16,17,18,19,20,21,22], whereas miR-20b and miR-200b exhibit decreased expression [16,17,18,19,20,21,22,23,24,25]. Hypoxia also induces miR-21 upregulation through Akt2 activation [26]. Among these miRNAs, miR-210, known as the “Master HRM,” consistently shows upregulation in response to hypoxia across various studies [27,28]. 

Communication within the tumor microenvironment relies heavily on extracellular vesicles (EVs) secreted by tumor cells, including exosomes (30–100 nm), microvesicles (100–1000 nm), and oncosomes (1–10 μm) [29]. EVs play crucial roles in this process by transferring functional molecules such as DNA, mRNAs, miRNAs, long non-coding RNAs (lncRNAs), proteins, lipids, and metabolites, along with their associated properties, between cells [30]. Sorting RNA and miRNAs into exosomes is tightly controlled, reflecting the cellular context and disease state. This selective packaging of RNA and miRNAs into exosomes facilitates the targeted delivery of messages to recipient cells, potentially influencing various cellular processes and contributing to disease progression, including cancer therapy resistance [31,32]. Remarkably, the specificity of this exosome cargo is such that analysis of exosomes secreted from brain tumor patients holds promise as a non-invasive diagnostic tool [33]. Recent evidence suggests that microvesicles may be used as biological carriers and offer significant advantages in targeted therapy [34].

Hypoxia enhances exosome release and alters their content, affecting intercellular communication. Hypoxic glioma-derived exosomes communicate between hypoxic gliomas and their TME [35]. For instance, miR-9 derived from glioblastoma EVs can impact vascularization through post-transcriptional modifications in endothelial cells [36]. Additionally, the hypoxic microenvironment stimulates gliomas to generate exosomes rich in miR-1246 and miR-10b-5p, which are delivered to normoxic glioma cells, promoting their migration and invasion [37]. Besides exosome-mediated communication, miRNAs can also be delivered from cell to cell via HDL association [38,39] and by binding to protein complexes like Argonaute-2 (Ago2) and nucleophosmin 1 (NPM1) [40,41,42].

Bevacizumab’s failure to significantly extend overall survival underscores the need for a deeper investigation into its limited effectiveness. Our previous study aimed to identify biomarkers for bevacizumab response to advanced precision medicine [43]. We discovered a robust correlation between bevacizumab response, tumor size changes, and serum levels of miRNAs 10b and 21 in glioblastoma patients. These miRNAs, involved in cancer and hypoxia pathways, could be vital in understanding the interaction between bevacizumab, hypoxia, and miRNAs.

This study aims to examine miR-21 and miR-10b expression dynamics in glioblastoma cells in vitro, offering insights into their reactions to hypoxia and secretion mechanisms. Additionally, it focuses on the specific influence of miR-10b on regulating VEGF, the pivotal regulator of angiogenesis.

## 2. Results

### 2.1. Hypoxia-Induced miRNA Expression

To explore if heightened serum levels of miR-10b and miR-21, noted in patients receiving bevacizumab as documented in our earlier study, are linked to increased tumor hypoxia, we subjected glioblastoma cell lines (A172, U87MG, U251) and human umbilical vein endothelial cells (HUVECs) to hypoxic conditions (1% oxygen) for 24 h. Given that VEGFA plays a pivotal role in angiogenesis and its expression is known to rise in response to hypoxia, it is a reliable marker [44,45]. Hence, we evaluated the expression levels of VEGFA in cells subjected to hypoxic conditions as a hallmark of hypoxia induction.

**VEGFA Expression Under Hypoxia:** Following hypoxia, we noted a significant increase in VEGFA expression (2–5 fold) after 24 h across all cell lines—A172 (*p* = 0.013), U87 MG (*p* = 0.004), U251 (*p* = 0.001), and HUVEC (*p* = 0.0004) compared to the respective cell line cultured under normoxic conditions—indicating the cells were indeed exposed to hypoxic conditions (Supplementary Figure S1).

**Figure 1:**
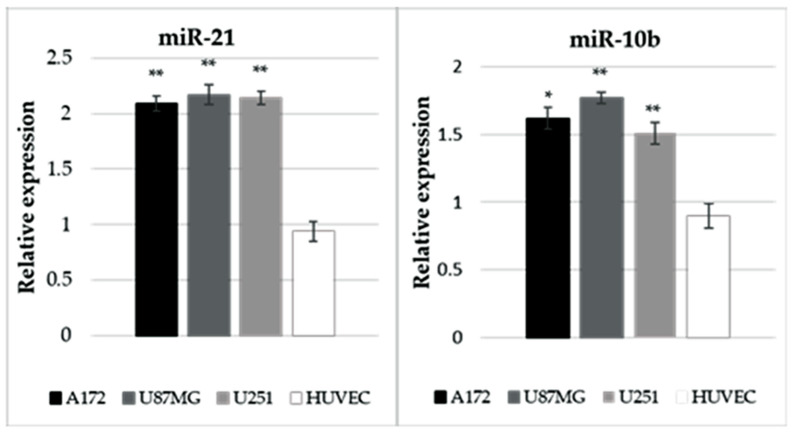
Impact of hypoxic conditions on miR-10b and miR-21 expression in glioblastoma and endothelial cell line. A172 (black bars), U87MG (gray bars), and U251 (light gray bars) glioblastoma cell lines, along with the endothelial cell line HUVECs (white bars), were exposed to 1% oxygen for 24 h, followed by RNA extraction for qPCR analysis. Quantification of both miRNAs was determined in each cell line relative to the respective cell line cultured under normoxic conditions. The asterisk indicates a significant increase under hypoxic conditions compared to normoxic conditions, with (*) denoting *p* < 0.05 and (**) denoting *p* < 0.01.

**miR-21 and miR-10b Expression:** Subsequently, miR-21 and miR-10b expression was quantified using the same RNA extracted from the hypoxic cells. The analysis revealed a significant increase (>1.5) in the expression of both miR-21 and miR-10b in glioblastoma cells under hypoxic conditions compared to normoxic conditions (miR-10b: *p* = 0.02 in A172, *p* = 0.003 in U87MG, and *p* = 0.001 in U251; miR-21: *p* = 0.0006 in A172, *p* = 0.004 in U87MG, and *p* = 0.004 in U251), while no such elevation was observed in HUVECs (*p* = 0.18 for miR-10b and *p* = 0.21 for miR-21) (Figure 1).

These findings prompted further investigation into the functional roles of these miRNAs under hypoxic conditions.

### 2.2. Role of miR-10b in Hypoxia

**miR-10b Expression Manipulation:** Recognizing the established role of miR-21 in modulating hypoxia [17], we further investigated the impact of miR-10b on hypoxia-related gene regulation by manipulating its expression in U87MG cells. To enhance its expression, we transfected U87MG cells with a plasmid over-expressing miR-10b (MDH1-PGK-GFP microRNA-10b) [46]. To inhibit its activity, we transfected a plasmid containing the target sequences of the miR into the cells (pBabe-puro-miR-10b sponge) [47], thus acting as a competitive inhibitor (“sponge”).

**Validation of miR-10b Transfection:** Initially, to confirm the effect of transfection on miR-10b expression, we analyzed miR-10b expression by qPCR in cells transfected with each plasmid. We observed that transfecting U87MG cells with the plasmid containing miR-10b increased its expression by 45-fold compared to cells transfected with the same plasmid lacking the miRNA (*p* = 1.46 × 10^−6^). Moreover, in cells double-transfected with both plasmids (miR-10b and sponge), the expression of miR-10b decreased by 9-fold relative to cells transfected with miR-10b alone (*p* = 2.23 × 10^−6^) (Supplementary Figure S2).

**VEGFA Expression Analysis:** To investigate the involvement of miR-10b in regulating the hypoxia-associated gene, VEGFA, we exposed cells transfected with miR-10b to hypoxic (1% oxygen) conditions for 24 h. Subsequently, we analyzed the expression of VEGFA in these cells compared to those transfected with miR-10b and incubated under normoxic conditions (17–20% oxygen).

**Figure 2:**
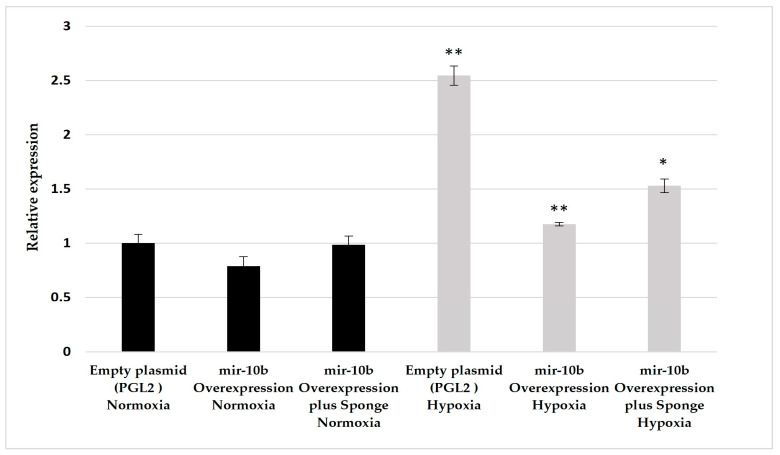
Relative quantification of VEGFA in U87MG cells following the overexpression of miR-10b under normoxic and hypoxic conditions. U87MG glioblastoma cells were transfected with various plasmids as indicated on the x-axis and incubated under either normoxic conditions (black bars) or hypoxic conditions (light gray bars). VEGFA expression was assessed by qPCR after 24 h of incubation. The asterisk indicates a significant expression following hypoxia induction in cells containing the empty plasmid compared to cells in normoxic conditions. Additionally, it denotes a significant expression under hypoxia in cells transfected with the miR-10b plasmid compared to cells transfected with the empty plasmid. Furthermore, it indicates a significant expression in cells under hypoxia co-transfected with the plasmid containing miR-10b and its competitive inhibitor compared to cells transfected with miR-10b alone. (*) represents *p* < 0.05 and (**) represents *p* < 0.01.

We observed a significant increase in VEGFA gene expression following hypoxia induction in cells containing the empty plasmid (*p* = 0.00121). However, overexpression of miR-10b led to a significant decrease in VEGFA expression under hypoxia (*p* = 1.8 × 10^−6^). This suggests that miR-10b overexpression hinders the upregulation of hypoxia-related genes, specifically VEGFA (Figure 2).

**Normoxic Conditions Observation:** Furthermore, we found that overexpression of miR-10b did not impact gene expression under normoxic conditions, indicating that it does not alter the basal levels of VEGFA. Notably, when we co-transfected the plasmid containing miR-10b with its competitive inhibitor (sponge), the expression of VEGFA significantly increased again (*p* = 0.02590), although not to the same levels as in the absence of the inhibitor. This result emphasizes the specific involvement of miR-10b in regulating the expression of VEGFA in hypoxic conditions (Figure 2).

While the results suggest a correlation between miR-10b expression and the hypoxia-related gene VEGFA, the mechanism underlying the decrease in gene expression after miR-10b elevation remains ambiguous. A literature review has not yielded a direct link between them. Moreover, analysis using two miRNA target prediction algorithms, namely miRDB—miRNA Target Prediction Database [48]—and Target-scan [49], did not reveal a target region of miR-10b in VEGFA.

The observed regulation of VEGFA by miR-10b under hypoxic conditions led us to further explore how hypoxia might influence the distribution of miRNAs in the glioblastoma cell line medium, particularly regarding exosomal signaling.

### 2.3. Hypoxia Induces Differential Distribution of miRNAs in Glioblastoma Cell Line Medium: Implications for Exosomal Signaling

**miRNAs Circulation Forms:** miRNAs are recognized to circulate in the bloodstream in diverse forms, including packaging within micro-vesicles such as exosomes or binding to protein or lipoprotein complexes like High-Density Lipoproteins (HDLs) [38].

**Size Exclusion Chromatography:** This technique facilitates the separation of distinct molecule populations based on their size characteristics. Larger particles, unable to permeate the pores, eluted earlier, while smaller particles with longer retention times eluted later, as illustrated in Supplementary Figure S3. Employing this analytical method, we fractionated the growth medium of U87MG cells under both normoxic and hypoxic conditions using size exclusion columns packed with porous resin particles approximately 75 nm in diameter. Subsequently, fractions 7–11 and 12–20 were concentrated, each representing molecules of varying sizes (Supplementary Figure S3).

**Figure 3:**
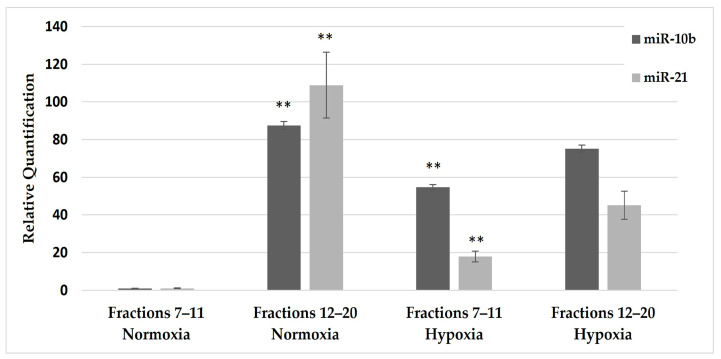
Quantification of miR-10b and miR-21 in size chromatography fractions from U87MG cell culture medium. U87MG cells were cultured under normoxic or hypoxic conditions for 24 h (as indicated), and their medium was processed using size exclusion columns. qPCR was then used to measure miR-10b (gray columns) and miR-21 (light gray columns) levels in each fraction. Exosomes, identified as large particles, are eluted in fractions 7–11, while proteins and molecules with a diameter smaller than 75 nm are eluted in fractions 12–20. The asterisk indicates a significant miR-10b and miR-21 quantification under normoxic conditions, particularly in the later eluted fractions (fractions 12–20), compared to the earlier eluted fractions (fractions 7–11). Additionally, it denotes a significant miRNA quantification following 24 h of hypoxia, specifically within the exosomal fractions (fractions 7–11) compared to the same fractions under normoxic conditions. (**) represents *p* < 0.01.

**miRNA Levels in Fractions:** We conducted a qPCR analysis to quantify the levels of miR-10b and miR-21 in each fraction. Under normoxic conditions, miRNA concentrations in the U87MG cell line medium were predominantly present in the later eluted fractions (fractions 12–20), particularly in the protein fractions compared to the earlier eluted fractions (fractions 7–11) (*p* = 0.00016 and *p* = 1.55 × 10^−6^, respectively). However, following 24 h of hypoxia, there was a notable increase of 55-fold and 18-fold in miR-10b and miR-21, respectively (*p* = 0.004 and 0.0003, respectively), specifically within the exosomal fractions (fractions 7–11) compared to the same fractions under normoxic conditions (Figure 3).

These results underscore the significant role of hypoxia in altering the distribution of miRNAs in glioblastoma cell line medium, with potential implications for exosomal signaling pathways and miRNA-mediated intercellular communication.

## 3. Discussion

Glioblastoma presents a formidable challenge in oncology due to its limited treatment options and poor prognosis. Despite the potential of bevacizumab, a monoclonal antibody targeting VEGFA to disrupt tumor angiogenesis [1], it faces challenges and limitations in treatment outcomes. Our prior research discovered elevated serum levels of miR-10b and miR-21 in glioblastoma patients undergoing bevacizumab treatment. These miRNAs hold promise as dependable biomarkers for assessing the response to bevacizumab treatment. This study aims to explore bevacizumab’s impact on miRNA expression, elucidate the role of miRNAs 10b and 21, particularly miR-10b, in hypoxia regulation, and investigate the mechanisms underlying miRNA secretion.

We investigated hypoxia’s impact on miR-21 and miR-10b expression by assessing their levels in glioma cell lines after 24 h of exposure to hypoxic conditions. Our findings demonstrated a significant increase in both miRNA levels across three glioma cell lines under hypoxic conditions. Interestingly, our experiments on HUVEC endothelial cells revealed no alteration in miRNA expression compared to tumor cells under hypoxia, suggesting that the elevation in miRNA levels in patients’ serum is likely attributable to increased expression in glioma cells rather than hypoxic endothelial cells. However, this result does not rule out the possibility that other cells, within or outside the TME, could contribute to the elevated miRNA levels in patients’ serum. It should be mentioned that artificially exposing HUVECs to VEGFA for 24 h increased the levels of miR-21 [50]. This inconsistency might be attributed to the differing methodologies employed between the studies. Specifically, in the other study, VEGFA was supplied externally, while in our research, the elevation in VEGFA expression was measured within the cells following exposure to hypoxia. Further investigations are warranted to validate these hypotheses and elucidate the precise mechanisms involved.

Hermansen SK et al. devised an image analysis-based co-localization method, assessing the miR-21 in situ hybridization signal in conjunction with immunohistochemical staining in astrocytomas. They revealed significant co-localization of miR-21 with proteins associated with the angiogenic genes HIF-1α and VEGFA, confirming a direct relationship between miR-21 and hypoxia [51].

In VEGFR2-luc transgenic mice implanted with breast cancer cells, miR-21 knock-down impaired angiogenesis by suppressing the HIF-1α/VEGF/VEGFR2 pathway. This evidence indicates that miR-21 expression is closely related to VEGF levels and displays a crucial functional interaction with VEGF in mediating angiogenesis. This further supports the possibility raised by our previous publication that the increase in miR-21 levels during bevacizumab treatment observed in our study may be a compensatory response to overcome therapy-induced inhibition of VEGF-mediated angiogenesis [52]. Combining anti-VEGF therapy with miR-21 inhibitors could offer a new avenue for enhancing the effectiveness of antiangiogenic therapy in patients with brain tumors.

Building on the established relationships between miR-21 and hypoxia, our study aimed to explore the role of miR-10b in hypoxia and its impact on VEGFA expression. We conducted experiments involving the artificial elevation of miR-10b expression in cells, followed by assessing VEGFA gene expression under both normoxic and hypoxic conditions.

Our results revealed that cells transfected with an empty plasmid exhibited increased VEGFA gene expression when exposed to hypoxia. However, overexpression of miR-10b inhibited this upregulation of VEGFA expression in response to hypoxia. This suggests that elevated levels of miR-10b exert a suppressive effect on hypoxia-induced VEGFA expression. Furthermore, co-transfecting the plasmid containing miR-10b with its competitive inhibitor (sponge) resulted in increased VEGFA expression, although not to the same extent as observed in the absence of the inhibitor. This highlights the specific involvement of miR-10b in regulating VEGFA. These findings are further supported by a study demonstrating that silencing miR-10b activity using a baculoviral vector encoding decoy miR-10b binding sites resulted in significant alterations in U87-2M1 cells, impacting cell growth, invasion, and angiogenesis [53].

Our observations revealed no notable changes in gene expression under normoxic conditions despite miR-10b overexpression. This suggests that miR-10b does not affect the basal levels of VEGFA expression when oxygen levels are within the normal range. This might imply that miR-10b serves as a sensitive regulator of VEGF, intervening only when VEGFA levels are increased to maintain VEGF concentrations within an optimal range. Maintaining VEGF levels within an optimal concentration range is crucial for facilitating mature blood vessel formation [53].

Additional research is required to elucidate the mechanisms governing the interaction between miR-10b and hypoxia-induced gene expression, especially regarding regulating VEGFA. Our findings suggest that hypoxia induces an upregulation of miR-21 and miR-10b expression.

Our investigation into the distribution of miRNAs in the medium of the U87MG glioma cell line under normoxic and hypoxic conditions revealed intriguing findings with potential implications for intercellular communication. Under normoxic conditions, miRNA concentrations were predominantly present in the cell culture medium’s later eluted fractions (12–20), particularly within the protein-enriched fractions. This distribution pattern suggests that miRNAs may primarily associate with proteins or other macromolecules in the extracellular environment under normal physiological conditions. However, during hypoxic conditions, we observed a significant shift in the distribution of miRNAs, particularly miR-10b and miR-21, towards the exosomal fractions (fractions 7–11) of the cell culture medium. Specifically, there was a notable increase of 55-fold in miR-10b and an 18-fold increase in miR-21 within these exosomal fractions compared to normoxic conditions. The increase in miRNA levels within exosomes during hypoxia suggests that hypoxia may trigger the selective packaging of miRNAs into exosomes, potentially facilitating cellular communication and adaptation to stressful conditions. These results align with studies demonstrating that cancer cells produce more exosomes under hypoxic conditions, thereby facilitating tumor intercellular communication at a distance, indicating the role of exosomes as vital regulators in hypoxic tumors [54,55].

King et al. exposed three different breast cancer cell lines to moderate (1% O_2_) and severe (0.1% O_2_) hypoxia, leading to a significant increase in the number of exosomes. They demonstrated that breast cancer cells release high levels of exosomes and miR-210 in hypoxic exosomes in an HIF-1α-dependent manner [56]. Similarly, in another study addressing the molecular mechanisms regulating exosomal shedding, Umezu et al. found that hypoxia-resistant multiple myeloma (HR-MM) cells produced more exosomes with a significantly higher expression of miR-135b compared to normoxic cells, indicating that the tumor-secreted exosomes could be induced by hypoxia. They showed that this exosomal miR-135b is transferred into endothelial cells by hypoxia-resistant multiple myeloma cells and targets HIF-1, thereby enhancing angiogenesis [57].

It has been suggested that RNA molecules carried by exosomes from glioma stem cells could impact the gene expression of endothelial cells, promoting angiogenesis. Researchers have identified eight candidate miRNAs that may play a role in angiogenesis mediated by EVs [36]. However, none of these miRNAs were enriched in the serum of glioblastoma patients after treatment with bevacizumab [43]. Thus, quantifying these miRNAs should be further explored in the circulation of patients with glioblastoma following treatment with bevacizumab.

Furthermore, studies have shown that when miR-9 is delivered to HUVECs via EVs derived from glioblastoma, its levels in HUVECs directly correlate with the formation of tubule-like structures [58]. These results underscore the dynamic nature of extracellular miRNA signaling and highlight the importance of considering cellular context and environmental factors in understanding their functional roles. This finding could explain our results, indicating a significant increase in miR-10b and miR-21 expression in glioblastoma cells following hypoxia but not in HUVECs. It can be speculated that, following hypoxia, the expression of miR-10b and miR-21 is heightened in glioblastoma cells. As demonstrated with miR-9, these miRNAs are then secreted via exosomes into the microenvironment, regulating gene expression in endothelial cells and potentially other surrounding cells.

In our previous clinical trial, we demonstrated an inverse correlation between the levels of miR-10b and miR-21 in circulation and tumor size as measured by MRI. The trial indicated that tumor growth was associated with a reduction in circulating miRNAs. We hypothesized that effective bevacizumab treatment and the resulting tumor hypoxia lead to an increased secretion of miRNAs into circulation. In this study, we aimed to test this hypothesis using cell lines to determine if hypoxia indeed triggers the expression of these miRNAs and whether normoxic conditions, which might correlate with the inefficacy of antiangiogenic treatment, result in reduced miRNA expression.

Based on the findings of the current study and the results of other studies, we propose the following mechanism: effective bevacizumab treatment occurs when the anti-VEGF agent binds to VEGFA, inhibiting blood vessel formation in the tumor microenvironment (TME) and increasing hypoxia due to reduced vessel density [1]. As we demonstrated, hypoxia triggers the upregulation of miR-10b and miR-21 expression, leading to their accumulation within exosomes in the cell culture medium. This may imply a mode of secretion of these miRNAs into the circulation of patients during tumor hypoxia.

Further research is needed to understand the molecular mechanisms underlying the selective packaging of miRNAs into exosomes during hypoxia and to explore the functional implications of exosomal miRNA transfer in glioma progression and therapeutic response.

The limitations of this study include the reliance on in vitro results, which may not fully reflect the conditions within the tumor and its TME in vivo. Additionally, the induced in vitro hypoxia may not precisely mimic the hypoxia present within the tumor and its TME. Further mechanistic studies demonstrating the roles of different actors in hypoxia regulation, such as HIFs, could enhance the study’s validity.

In summary, hypoxia-induced miRNA changes and their potential as treatment response biomarkers were explored. The findings of our previous study [43] and the current findings could enhance diagnosis and treatment in brain tumor patients.

## 4. Materials and Methods

### 4.1. RNA Extraction

RNA extraction was conducted with the MasterPure ™ Complete DNA and RNA Purification Kit from Epicenter (Epicentre, Madison, WI, USA). We extracted RNA from the cells according to the manufacturer’s protocol for cell sample RNA extractions. We added 1 µL of glycogen to improve the extraction. At the end, we diluted the RNA in 20 µL of ultra-pure DNase/RNase-free water.

We analyzed the nature and concentration of RNA by nanodrop.

### 4.2. CDNA and Quantitative Polymerase Chain Reaction (qPCR)

cDNA was produced from 0.2 µg total RNA with a qScript cDNA Synthesis Kit (Quanta Biosciences, Gaithersburg, MD, USA), according to the manufacturer’s instructions. For analyzing miRNA expression, cDNA was produced from 10.5 µL total RNA with qScript MiRNA cDNA Synthesis Kit (Quanta Biosciences, Gaithersburg, MD, USA).

### 4.3. Real-Time Polymerase Chain Reaction Amplification and Relative Quantification

Real-time PCR amplification and relative quantification were analyzed with StepOne real-time RT PCR (Life Technologies, Carlsbad, CA, USA). The reaction mix included 1 μL cDNA, 300 nmol/L of each of the following primers (Syntezza, Jerusalem, Israel), and 5 μL of SYBR green mix (Perfecta Syber Green Fast Mix ROX, Quanta Biosciences, Gaithersburg, MD, USA) in a total of 10 μL volume. The fold changes of the target mRNAs were normalized to HPRT and TUBB. The experiment was repeated three times in triplicate, and the results are presented as the mean ± SD.

Primers: HPRT fwd: 5′: AGATGGTCAAGGTCGCAAGC3′

HPRT rev: 5′: CATATCCTACAACAAACTTGTCTGGAA3′

TUBB fwd: 5′: CATACATACCTTGAGGCGAGCA3′

TUBB rev: 5′: TCACTGATCACCTCCCAGAACTT3′

VEGFA fwd: 5′: GAGTCCAACATCACCATGCAGAT3′

VEGFA rev: 5′: GAAGCTCATCTCTCCTATGTGCTG3′

The miRNA amplification was carried out using primers for the relevant miRNAs, and forward primer sequences appear below. Since the RNA was polyadenylated, oligo dT was used as the reverse primer. The fold changes of the target miRNAs were normalized to RNU6.

RNU6: GUGCUCGCUUCGGCAGCACAUAUACUAAAAUUGGAACGAUACAGAGAAGAUUAGCAUG

hsa-miR-10b-5p: UACCCUGUAGAACCGAAUUUGUG

hsa-miR-21-5p: UAGCUUAUCAGACUGAUGUUGA

The experiment was repeated three times in triplicate, and the results are presented as the mean ± SD.

### 4.4. Cell Culture

Cell lines A172, U87MG, and T98G (glioblastoma) were obtained from the American Type Culture Collection (Manassas, VA, USA). A172 and U87MG cells were cultured in Dulbecco’s modified Eagle’s medium (DMEM) supplemented with 4 mmol/L L-glutamine, 100 units/mL penicillin, 100 μg/mL streptomycin, and 10% FBS (Biological Industries, Beit Haemek, Israel). The T98G cells were cultured in Eagle’s minimum essential medium supplemented with L-glutamine, penicillin, streptomycin, and FBS. HUVECs were generously provided by Prof. Ofra Benny’s Laboratory at the Hebrew University and cultured in PeproGrow™ MacroV (Peprotech, Cranbury, NJ, USA) supplemented with Growth supplement Macro-V (Peprotech) and 100 units/mL penicillin, 100 μg/mL streptomycin. The cells were grown in flasks coated with 1% gelatin. All cells were maintained in a humidified incubator at 37 °C in 5% CO_2_. The cells were passaged twice a week to prevent over-confluency and to maintain their proliferative capacity using the following subculturing procedure: the culture medium was aspirated, and the cells were washed with phosphate-buffered saline (PBS) to remove the residual medium. Trypsin-EDTA solution A (Biological Industries, Beit Haemek, Israel) was added to detach the cells from the flask surface. After sufficient detachment, the trypsinization was stopped by adding an equal volume of complete growth medium. The cell suspension was then centrifuged, and the cell pellet was resuspended in a fresh growth medium. The cells were seeded into new culture flasks at the desired density. The cell cultures were regularly tested for mycoplasma (Mycostrip, Invivogen, Toulouse, France) contamination to ensure the authenticity and purity of the cell lines.

### 4.5. Hypoxia

A total of 2 × 10^5^ cells were plated in 6-well plates (Thermo Fisher Scientific Inc., Waltham, MA, USA) and allowed to attach overnight. One plate remained in the incubator under normal conditions (control), and one was placed in a hypoxia-modular incubator chamber (MIC 101—Billups Rothenberg Inc., San Diego, CA, USA). The hypoxia chamber was connected for one minute and a half to a gas mixture of 94% nitrogen, 5% CO_2_, and 1% oxygen. Using an oxygen analyzer, we sealed the chamber when the oxygen level reached 1% and placed the plate in an incubator for 24 h. We then harvested the cells and analyzed miRNAs and gene expression.

### 4.6. Extraction of Plasmids from Bacteria

MDH1-PGK-GFP microRNA-10b was a gift from Bob Weinberg (Addgene plasmid # 16070; https://www.addgene.org/; RRID: Addgene_16070) [46]. pBabe-puro-miR-10b sponge was also a gift from Bob Weinberg (Addgene plasmid # 25043; https://www.addgene.org/; RRID: Addgene_25043) [47].

Bacteria with plasmid DH1-PGK-GFP expressing miRNA-10b and with the plasmid pBabe-puro-miR-10b sponge were seeded on LB agar plates containing ampicillin at a concentration of 50–100 µg/mL and grown at a temperature of 37 °C, overnight (until colonies containing the plasmid were formed). Then, a colony was lifted and transferred to a 15 mL test tube containing about 5 mL LB broth with 50–100 µg/mL ampicillin, and the test tube was incubated for one day with shaking at 37 °C. The plasmids were extracted using Qiagen’s Compact prep plasmid maxi kit, according to the manufacturer’s protocol. The amount of DNA obtained was quantified using nanodrop, and the DNA was run in TAE gel to check the quality of the plasmid.

### 4.7. Temporary Cell Transfection (Overexpression/Inhibition of miR-10b)

A total of 2 × 10^4^ U87MG cells were plated in 24-well plates and allowed to attach overnight. Then, the cells were transfected using the jet PEI reagent (Polyplus, Illkirch-Graffenstaden, France) with the MDH1-PGK-GFP MiRNA-10b plasmid, pBabe-puro-miR-10b sponge, and a blank plasmid for control. We added 100 μL of reagent and DNA mixture per the manufacturer’s protocol. The cells were incubated for 24 h and then harvested for miRNAs and gene expression analysis.

### 4.8. Extraction of Exosomes from Cell Medium

We extracted exosomes from the cell medium using the qEV original Size Exclusion Column (Izon Science, Oxford, UK), following the manufacturer’s protocol. We chose this size-exclusion chromatography (SEC) method because it has been demonstrated in numerous studies to be fast and reliable. The exosome population isolated using this method is homogeneous in size, morphology, and protein composition [59,60].

Briefly, 500 µL of filtered medium (22 µm) was loaded onto the column. Filtered PBS was added once the medium entered the column, and 500 µL fractions were collected. The column contains resin with pores of 75 nm in size. Proteins and small molecules are inhibited from entering the pores, while larger molecules are released first upon the addition of the liquid. Consequently, the initial fractions contain large entities, such as exosomes and vesicles, while later fractions contain proteins and small molecules. We combined fractions into three groups: Fractions 7–11, Fractions 12–20, and Fractions 21–30. Each group of fractions contains molecules of different sizes. The fractions were concentrated using Amicon Ultra 15 mL 100 K (Merck, Darmstadt, Germany) filters to obtain a final volume of approximately 150–200 µL. RNA was then extracted from these three fractions to analyze the representation of different miRNAs in each fraction.

### 4.9. Statistical Analysis

We employed a statistical method described by Hien Fuh Ng et al. [61]. Utilizing the spreadsheet provided in their manuscript, we conducted our statistical analyses accordingly. For statistical comparisons, we used a one-tailed test when comparing the same cell line under different conditions, such as normoxia and hypoxia. For comparisons between transfected and non-transfected cells, we employed a two-tailed test.

The analysis in this study compared two selected groups as indicated, using either a two-tailed or one-tailed Student’s *t*-test, following the method outlined in the article by Hien Fuh Ng et al. [61]. A *p*-value of <0.05 was considered statistically significant.

## 5. Conclusions

In glioblastoma treatment, bevacizumab’s efficacy is hindered by hypoxia-induced challenges. Our previous study demonstrated upregulation of miR-10b and miR-21 in the serum of patients with high-grade gliomas following antiangiogenic treatment, with a significant negative correlation between these MiRNAs’ expression and changes in tumor volume measured by MRI. This study reveals significant increases in miR-21 and miR-10b expression in glioma cells under hypoxic conditions, implicating their roles in response to low oxygen levels and angiogenesis regulation. MiR-21’s association with hypoxia, VEGFA regulation, and miR-10’s potential as a therapeutic target highlight their significance in glioblastoma progression. Moreover, the shift towards exosomal packaging of miR-21 and miR-10b during hypoxia suggests a mechanism for intercellular communication under stress conditions. These findings suggest that targeting miRNAs in combination with anti-VEGFA therapy could enhance treatment efficacy by disrupting angiogenesis and overcoming resistance mechanisms. However, further research is needed to validate these findings and explore their clinical implications for personalized glioma treatments.

## Data Availability

The original contributions presented in the study are included in the article/supplementary material; further inquiries can be directed to the corresponding author/s.

## Supplementary Materials

The following supporting information can be downloaded at: https://www.mdpi.com/article/10.3390/ijms25147984/s1.
