# Supplementary material for: Investigating Expression Dynamics of miR-21 and miR-10b in Glioblastoma Cells In Vitro: Insights into Responses to Hypoxia and Secretion Mechanisms
Source: Int J Mol Sci. 2024 Jul 22;25(14):7984. doi: 10.3390/ijms25147984 (PMC11277016; doi:10.3390/ijms25147984)
Supplement: Supplementary file 1 [file ijms-25-07984-s001.zip › ijms-3100847-supplementary.pdf]

# Investigating Expression Dynamics of miR-21 and miR-10b in Glioblastoma Cells in Vitro: Insights into Responses to Hypoxia and Secretion Mechanisms

**Supplementary figures.**

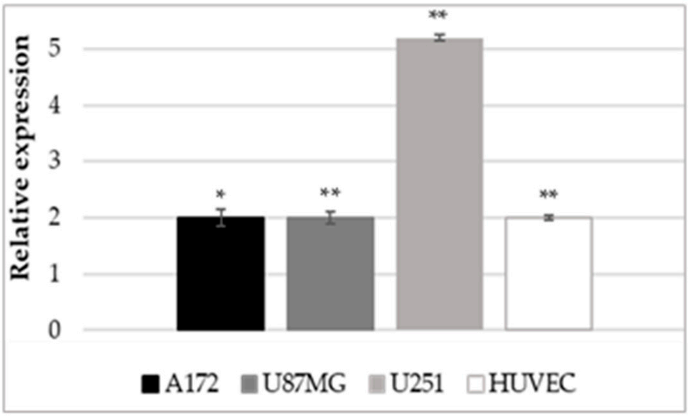


**Supplementary Figure S1:** RNA Relative quantification of VEGFA following 24 hours of hypoxia in glioblastoma and endothelial cell line. A172 (black bars), U87MG (grey bars), and U251 (light grey bars) glioblastoma cell lines, along with the endothelial cell line HUVEC (white bars), were subjected to 1% oxygen incubation for 24 hours, followed by RNA extraction for Quantitative polymerase chain reaction (qPCR) analysis. Expression levels of VEGFA in each cell line are compared to the respective cell line cultured under normoxic conditions. The asterisk indicates a significant increase under hypoxic conditions compared to normoxic conditions, with (*) denoting p<0.05 and (**) denoting p<0.01.

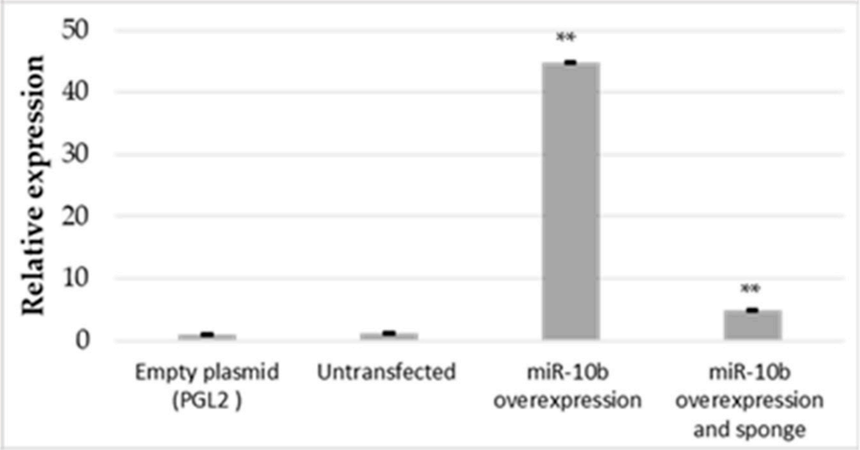


**Supplementary Figure S2:** Relative quantification of miR-10b in U87MG glioma cells 24 h after transfection with various plasmids.

U87MG glioblastoma cells were transfected with various plasmids as indicated on the x-axis, and miR-10b expression was evaluated by qPCR 24 hours after transfection. Quantification was determined relative to the expression of miR-10b in cells transfected with the control plasmid (empty PGL2). Additionally, miR-10b levels were measured in untransfected cells to verify that the empty plasmid alone did not influence miRNA expression. The asterisk indicates a significant increase post-transfection with the plasmid containing miR-10b compared to cells transfected with the same plasmid lacking the miRNA. Additionally, when comparing with cells double-transfected with both plasmids (miR-10b and Sponge) relative to cells transfected with miR-10b alone, (*) represents $p<0.05$ and (**) represents $p<0.01$.

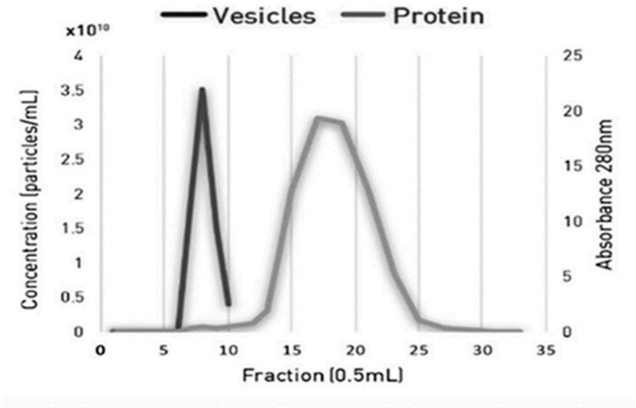


**Supplementary Figure S3:** The typical extraction profile of IZON size column. Proteins (grey line) are eluted in the latter fractions compared to vesicles released from the column in the first fractions (black line).
